# People conform to social norms when gambling with lives or money

**DOI:** 10.1038/s41598-023-27462-1

**Published:** 2023-01-16

**Authors:** Yueyi Jiang, Przemysław Marcowski, Arseny Ryazanov, Piotr Winkielman

**Affiliations:** 1 Department of Psychology, University of California San Diego, La Jolla, CA USA; 2 Swartz Center for Computational Neuroscience, University of California San Diego, La Jolla, CA USA; 3 Department of Psychology, SWPS University of Social Sciences and Humanities, Warsaw, Poland

**Keywords:** Human behaviour, Health policy

## Abstract

Many consider moral decisions to follow an internal “moral compass”, resistant to social pressures. Here we examine how social influence shapes moral decisions under risk, and how it operates in different decision contexts. We employed an adapted Asian Disease Paradigm where participants chose between certain losses/gains and probabilistic losses/gains in a series of moral (lives) or financial (money) decisions. We assessed participants’ own risk preferences before and after exposing them to social norms that are generally risk-averse or risk-seeking. Our results showed that participants robustly shifted their own choices towards the observed risk preferences. This conformity holds even after a re-testing in three days. Interestingly, in the monetary domain, risk-averse norms have more influence on choices in the loss frame, whereas risk-seeking norms have more influence in the gain frame, presumably because norms that contradict default behavior are most informative. In the moral domain, risk-averse as opposed to risk-seeking norms are more effective in the loss frame but in the gain frame different norms are equally effective. Taken together, our results demonstrate conformity in risk preferences across contexts and highlight unique features of decisions and conformity in moral and monetary domains.

## Introduction

In our daily lives, we frequently experience and discuss moral events. For example, in one study, when people were randomly prompted by their smartphone to respond about the events they encountered in the past hour, 29% of the reported events were interpreted as moral or immoral acts with people being involved in the acts either as an agent or a target, or hearing about them from others^1^. Traditionally, judgements about moral events have been thought to reflect an internal “moral compass”. This view posits that moral sense is a core aspect of human nature, as stated by Immanuel Kant^2^: “*Two things fill the mind with ever new and increasing admiration and awe, the more often and steadily we reflect upon them: the starry heavens above me and the moral law within me*”. This view is also aligned with trait essentialism, the belief that specific characteristics of an individual, such as morality, remain immutable and stable^3^.

However, research shows that moral values are *shaped by* social norms over time through various developmental stages from infants to adults, suggesting that morality is not immutable^4^. This fits with Bandura’s social learning theory that children observe and adopt the behaviors and attitudes of others^5^. Specifically, children’s moral judgment responses can be modified through adult modeling cues and social reinforcement^6^. This suggests that the development of moral judgement is closely associated with our abilities to identify a shared social norm, detect deviations between own behavior and the norm, and correct behavior to adapt to the social norm^7,8^.

Indeed, social norms are a powerful influence in prompting an individual to adopt others’ beliefs and preferences, in both non-moral and moral domains. In the non-moral domain, studies have shown that people conform to others’ perceptual judgements^9^, political views^10^, ratings of attractiveness^11,12^, and even opinions about selves^13^. In the moral domain, certain moral preferences may be especially vulnerable to social influence. For example, one study found that after participants learned about the choices of a person who prefers highly punitive measures, they enhanced their own endorsement of punishment to match the feedback learned from the person^14^. This matching behavior implies the important role of social norms in forming people’s punitive values. Another study, using the classic Solomon Asch Paradigm, found that participants conformed to confederates’ judgement about whether an action was morally permissible^15^. There is also evidence showing that charitable giving is influenced by social information about others’ behaviors^16,17^. These findings suggest that moral preferences are highly sensitive to social norms, which challenges the view that people’s moral decisions and judgements are primarily the product of an internal “moral compass”.

Despite considerable scientific interest in exploring conformity in the moral domain, research is scarce on whether and how social norms influence moral decisions that require trading off potential losses and gains in situations of uncertainty. Note that these decisions are involved in many real moral scenarios. These may include a juror reaching a guilty verdict despite uncertain evidence, a criminal choosing a profitable but risky immoral activity, a military commander ordering a strike on an enemy with some probability of harming innocent victims, or a doctor deciding on how to allocate medical resources and personnel to patients during a pandemic. Here, we examine whether a social conformity effect is present in moral decisions involving risk. When doing this, we assume a broad definition of "moral decisions" where individual choices have harmful and helpful consequences for others and are at least partially based on beliefs what constitutes proper behavior^18^. Also, we focus on the extent to which conformity effects occur in different types of norms such as risk-averse or risk-seeking norms, and different framings of moral issues such as gains and losses of lives.

Importantly, as philosophers and legal scholars have argued^19,20^, these moral decisions (and dilemmas) can be understood using classic psychological tasks in which individuals are asked to choose between a sure outcome and a probabilistic outcome that involve gains or losses of lives. One classic paradigm is the “Asian Disease Problem” (ADP)^21^, in which participants chose between a certain outcome (e.g., 200 lives lost for sure) and a risky option (e.g., 33% loss of 600 lives). Originally worded in terms of medical decisions, the ADP has been adapted to research on morality, as in the pioneering work of Ginges and Atran (2011) on risky versus certain military and diplomatic options^22^. It is worth noting, though, their studies used a design where participants made only one single choice between *two* options, and thus did not capitalize on the possibility to study choices in a parametric way (across a range of values). The parametric aspect (multiple decisions across a range of outcomes) is key for understanding when and what decisions are influenced by norms. Bearing this limitation in mind, we adapted this paradigm to enable the assessment of social influence on moral preferences under risk by parametrically varying the numerical estimates of gains and losses. This approach not only bridges our understanding of social conformity and risk preferences, but also offers a parametric, computationally tractable way to assess *how* conformity affects risk preferences.

Our version of the “Asian Disease Problem” (ADP) presents a hypothetical life-death dilemma in which individuals choose between a certain option that leads to definitely losing (or definitely saving) 10 lives, and a risky option that leads to a 50% chance of losing (or saving) some number of lives. In ADP, we can parametrically vary the *magnitude* of outcomes under a set probability. This allows us to determine individuals’ decision parameters such as the level of risk aversion. It also allows for testing the impact of gain or loss framing, a critical determinant in decisions^21^. As specified by Prospect Theory, when the two options have the same expected value, people tend to be risk-seeking (prefer the gamble over the certain option) in the domain of losses and risk-averse (prefer the certain over the risky option) in the domain of gains. These differing risk attitudes reflect diminishing sensitivity to larger amounts (so, the marginal effects of a larger gain, or a larger loss are less impactful than those of a smaller gain or a smaller loss). To assess conformity in ADP, we use a three-phase protocol that parallels the design used by FeldmanHall et al. (2018)^14^. In the first baseline phase, participants’ own risk preferences are assessed. In the second learning phase, participants learn other people’ preferences by observing their choices. In the final transfer phase, participants’ own preferences are assessed again. This protocol allows us to measure changes in participants’ behaviors following observation of the group behaviors.

Our adapted ADP and the three-phase protocol can not only be used to assess decision-making and conformity in moral decisions, but also to conduct a comparison between moral and non-moral domains—a subject of important debates in psychology and philosophy^23–26^. First, note that moral and non-moral decisions differ in many ways. For example, moral decisions may involve more deontological considerations whereas non-moral decisions may involve more utilitarian considerations^25^. In another example, moral as opposed to non-moral decisions can be more involved in maintaining a positive self-image (e.g., “I am an empathetic person who helps others”)^27^. Turning to social influence, social psychological theories suggest that conformity in moral and non-moral domains arise from different considerations. One such difference is related to two different categories of social norms. Note that descriptive norms refer to people’s beliefs about what most others do, whereas injunctive norms refer to people’s beliefs about what others approve or disapprove^28^. In moral situations, norms may come from social information about what others do (descriptive norms) but they also come with a sense of obligations of what should be done (injunctive norms)^29^. On the other hand, norms in the non-moral domain are more likely to be descriptive as they do not necessarily involve the perception of approval or appropriateness of certain behaviors. Additionally, literature suggests that when people make moral decisions they are concerned with securing good moral reputation in others’ eyes^27,30^. Related to this, motivations for conformity may be unique in the moral domain and stem from reciprocal expectations concerning other agents^31^. All these considerations suggest that it is important to compare moral and non-moral decisions and their relative sensitivity to social norms. We will return to these issues in the discussion.

To summarize, the present research addresses three aims. First, we examine whether people shift their decisions to align with the group’s preferences. If their own preferences in the transfer phase differ from those in the baseline phase, it would indicate that preferences for decisions involving risk are malleable. Second, we examine whether conformity operates differently across contexts. Do conformity effects depend on the type of the social norm (i.e., risk-averse and risk-seeking norms), and framing of the decision context (i.e., gains and losses)? Finally, we seek to understand whether decision-making and group conformity are unique in the moral domain compared to non-moral domain, given the difference in the motivations to conform and in the types of the decisions. Thus, our research looks at conformity in decisions about lives and also in decisions about money.

## Overview of the studies

We present two studies that explored social conformity on decisions about potential gains or losses in the moral or the monetary (non-moral) domain. Experiments 1 examined whether people would shift *moral* risk preferences towards the group norms that are either generally risk-averse or risk-seeking. Following a similar study set-up to Experiment 1, Experiment 2 tested whether group preferences influenced participants’ risk preferences in *monetary* decisions. Both experiments also assessed the influence of social norms after a three-day delay. Using this design, we aim to examine people’s tendency to conform to risk-averse and risk-seeking social norms in relation to both gain and loss framing, and decision-making in moral and monetary domains.

## Method

The current study was reviewed and approved by the University of California San Diego Institutional Review Board, approval #181176. All experiments were performed in accordance with the relevant guidelines and regulations. In each experiment, we employed a Time1-Time2 design where participants were invited to two separate study sessions. Participants’ risk preferences were assessed at a day 0 session, and subsequently at a day 3 follow-up session. All study participants provided informed consent.

For each experiment, we fitted two mixed effects logit models to participants’ choices whether to gamble or not. The first model was fitted using data from all participants who completed the day 0 session, and the second model was fitted using the data collected from those who completed both sessions. Unless stated otherwise, all reported effects and comparisons of gambling rates were derived from model estimates. In all post-hoc comparisons a multiplicity adjustment was applied using the multivariate *t* distribution with the same covariance structure as the estimates to determine the adjustment. All analytical procedures were performed in the R computational environment^32^.

### Experiment 1

Experiment 1 investigated whether observing a group’s preferences would influence people’s personal risk preferences in scenarios involving animal lives. We used both *gain* and *loss* framings of a modified ADP—the Moral Gamble Task to examine the generality of the conformity effect. Any possible gain–loss asymmetries are also informative about the underlying decision mechanisms^21^. More importantly, they are key for assessing a unique moral dimension of these judgments. Specifically, philosophers of morality have argued that people engage moral reasoning more in scenarios associated with losses than in gains, presumably because they involve different obligations or duties^19,23^.

In the Moral Gamble Task, we chose animal instead of human lives in our decision scenarios for the following reasons. First, moral decisions about animal lives feel personally realistic to our participants. In our sample (college undergrads), most participants do not have past personal experiences with meaningful decisions about human lives. However, they are more likely to encounter decisions regarding animal lives because of ethical food choices or pet ownership. Moreover, using animals in our decision scenarios allows us to create a plausible (and true) cover story that participants’ responses would be informative to discussions about animal welfare and conservation efforts. Second, recent research suggests that people have the tendency to care and feel empathy for animals like the way they do for other humans^33^. In fact, Hsee and Rottenstreich (2004) have used pandas as affect-rich stimuli to explore the nature of decision-making processes^34^.

We hypothesized that individuals exposed to a risk-seeking or risk-averse group’s preferences would later resemble the group’s behaviors in their own choices. Furthermore, we hypothesized that the effect of conformity would persist at least three days as suggested by previous conformity studies^35,36^. This set-up allows us to address if conformity effects on decisions persist after a time delay, and when participants are re-tested in a separate setting. This addresses the theoretical question of whether conformity behavior reflects transient situational pressures (normative conformity) versus informational factors that lead to norm internalization^37^. This Time1-Time2 design (along with data from the debriefing) reduces a potential concern that participants temporarily change how they respond due to perception of demand characteristics from the experimenter.

### Participants

471 participants (249 females, mean age = 20.15 years, range = 18–35 years, *SD* ± 3.47 years) were recruited from the UCSD online psychology subject pool for course credits. Following a 2 × 2 between-subject design, participants were randomly assigned to a gain or loss frame of the Moral Gamble Task and were randomly assigned to risk-averse or risk-seeking group norms. Participants were invited to complete two separate sessions, three days apart (see study details in the “Testing Procedure” section). Both sessions took between 30 min to 1 h to complete, and participants received one course credit for each session. To ensure comprehension of the task, we excluded 104 individuals in the first session (Day 0) who failed comprehension checks (i.e., answered two or more questions wrong out of four questions). Seven more individuals were excluded because they gave the same answer to all questions in the behavioral task, leaving a sample of 360 participants in the first session. On day 3, 320 participants came back for the second session. From the sample, we first excluded individuals who failed attention checks at the first (Day 0) session or gave the same answers, leaving 199 participants. Out of those 199, 61 failed the separate comprehension checks in the second session, leaving 138 participants who completed both sessions and passed separate comprehensions at both sessions.

In the analyses presented below, we first look at behavior of participants in the first session which we refer to as day 0 (D0). This allows us to establish the basic conformity effects. However, since we are interested in investigating the persistence of conformity over time, we also look at behavior during the follow-up session on day 3 (D3) and compare it to behavior on day 0. As mentioned, to do this, we take participants who completed both D0 and D3 sessions, and passed comprehension tests in both, leaving us a sample of 138 participants (92 female; age *M* = 19.73, *SD* = 1.67). Importantly, we found no difference between those participants who did both sessions and those who only completed the day 0 session. As presented in SI, the participants remaining for both sessions did not differ from those who dropped out in terms of choice responses, data quality (as indicated by participants’ sensitivity to EV), and conformity effects during day 0 session.

### Behavioral task

To study the impact of framing and social norms on conformity effects, we adapted ADP to create the Moral Gamble Task to probe the change of participants’ moral preferences. On each trial of the task, participants were asked to choose between a certain option and a gamble option in a hypothetical scenario. We held constant the value of the certain option at 10 and the probability of the gamble option at 50% but parametrically varied the gamble value of the gamble option from 10 to 30. Before the main procedure, all participants were presented with the task description either in gain or loss frame:

Welcome to the Moral Game! The purpose of this research is to understand people’s moral preferences in relation to decisions on animal welfare. In addition to providing your own opinions, you will be asked to guess how the majority people would respond to various decisions.

Imagine that a wildlife refuge is preparing for the outbreak of an unusual infectious disease, which is expected to kill many pandas. Two alternative programs to combat the disease have been proposed. Both programs have different consequences for different groups of pandas. Assume that the exact scientific estimates of the consequences of the programs are as described in each scenario. On each trial, you will be asked to choose between TWO options: (1) Gamble Option—50% chance of losing (saving) a number of pandas and (2) Certain Option—certainly losing (saving) 10 pandas. The number of pandas for the Gamble Option will vary across the trials. The number of pandas for the Certain Option will stay the same across the trials. You will be asked to indicate your own choice and to guess what the majority people choose.

After viewing the instruction, participants were asked four comprehension questions regarding the details of the task (e.g., choice types and options).

### Testing procedure

**Figure 1:**
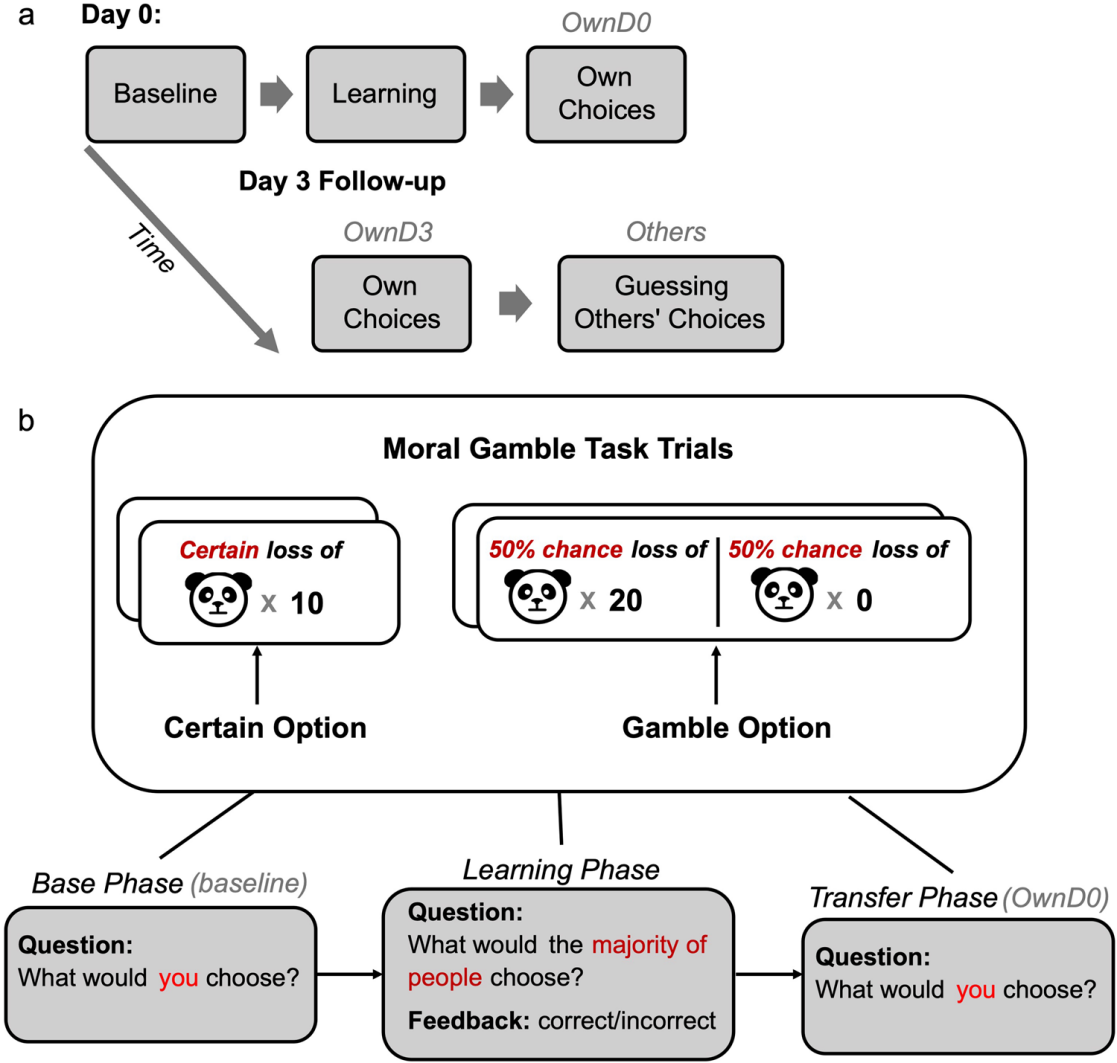
General study procedure. (**a**) At day 0 session, participants completed baseline, learning and transfer phases. At day 3 follow-up session, participants indicated own choices again (same decision scenarios as in the baseline) and guessed the group’s choices (same decision scenarios as in the learning phase). (**b**) In each trial of the moral gamble task, participants chose between a certain option and a gamble option for own choices. In gray, we show the questions asked in the task trials for each phase at day 0. In the learning phase, participants were given feedback regarding their guessing results.

Day 0 and day 3 follow-up sessions consist of different task trials and phases (see Fig. 1 for a general study procedure). Specifically, the testing procedure at day 0 consists of three phases. In the first and baseline phase, participants were asked to indicate their own choices in each of 11 different scenarios (gamble values ranged from 10 to 30 with increments by 2). In the second and learning phase, participants were tasked with guessing (with feedback) a group’s choices in 21 different scenarios (gamble values ranged from 10 to 30 with increments by 1). In the third and transfer phase, participants indicated their own choices again for the same scenarios as in the baseline phase. The testing procedure at day 3 consists of two phases: participants first reported their own choices for the same scenarios as in the day 0 baseline, and then guessed others’ choices for the same scenarios as in the day 0 learning phase but with no feedback. The scenarios for both day 0 and day 3 were presented in a randomized order with each of them repeating 4 times. In the rest of the paper, we will also use the term *OwnD0* to refer to the transfer phase, and *OwnD3* to represent the phase where participants indicated their own choices at day 3.

One critical component to elicit social conformity effects in the testing procedure is to get the participants to learn about the group’s risk preferences. This occurs in the day 0 learning phase where we provided a positive or negative feedback for a correct or incorrect guess on group’s choice. We constructed the feedback following a probabilistic schedule. Specifically, we systematically adapted the average gambling rates from a pilot study to be more risk-averse (i.e., 20% above group gambling rate) and more risk-seeking (20% below group gambling rate) to establish risk-averse and risk-seeking group norms (see SI and Fig. S-3 for additional details).

## Results and discussion

We fitted a mixed effects logit model to participants’ choices with explanatory variables including gamble value, experimental phase (*baseline*; *OwnD0*, own choices at day 0 after learning about group norms; *OwnD3*, own choices at day 3; *others*, guessing of others’ choices at day 3), norm condition (*risk-averse* or *risk-seeking*), and framing (*gains* or *losses*)—with random subject-level intercepts. Two separate models were fitted to investigate basic conformity effects and their persistence using the bigger sample on day 0, and the smaller sample of participants who completed both sessions, respectively.

First, we tested a few basic properties of our data collected from those who completed the day 0 session: (1) *baseline differences*: whether our randomization was successful (there were no baseline differences between groups later assigned to different experimental conditions); (2) *sensitivity to gamble values*: whether participants were sensitive to the expected values of the gamble option; (3) *risk tendencies*: whether participants were overall *risk-seeking* or *risk-averse*; and (4) *effects of framing*: whether participants were *risk-averse* in gains and *risk-seeking* in losses, consistent with Prospect Theory. Then, we examined: (5) *conformity effects*: whether participants conformed to the group norm; and (6) *duration of conformity*: whether conformity effects persist after a time delay using data from those who completed both sessions.

1. *Were there any baseline differences between groups later assigned to the experimental conditions?* Our randomization was successful. As expected, responses in the *baseline* did not differ between the experimental conditions in gains (*c* = 0.003, 95% CI [−0.08, 0.09]; *t* = 0.07; *p* = 0.943) or in losses (*c* = −0.02, 95% CI [−0.18, 0.14]; *t* = −0.30; *p* = 0.776), as evidenced by a model-based test of contrasts. This is important because selective attrition (e.g., due to failed comprehension checks) could potentially violate random assignment.
2. *Were participants sensitive to the expected values?* Yes, when the number of lives in the gamble option increased (while the certain option always remains at 10), participants’ choice of the gamble option increased in the gain frame and decreased in the loss frame (see Fig. 2a). This is evidenced by a significant marginal effect of gamble value in gains (*β* = 0.86, 95% CI [0.84, 0.89]; *p* < 0.001) as well as in losses (*β* = −0.94, 95% CI [−0.97, −0.91]; *p* < 0.001).

**Figure 2:**
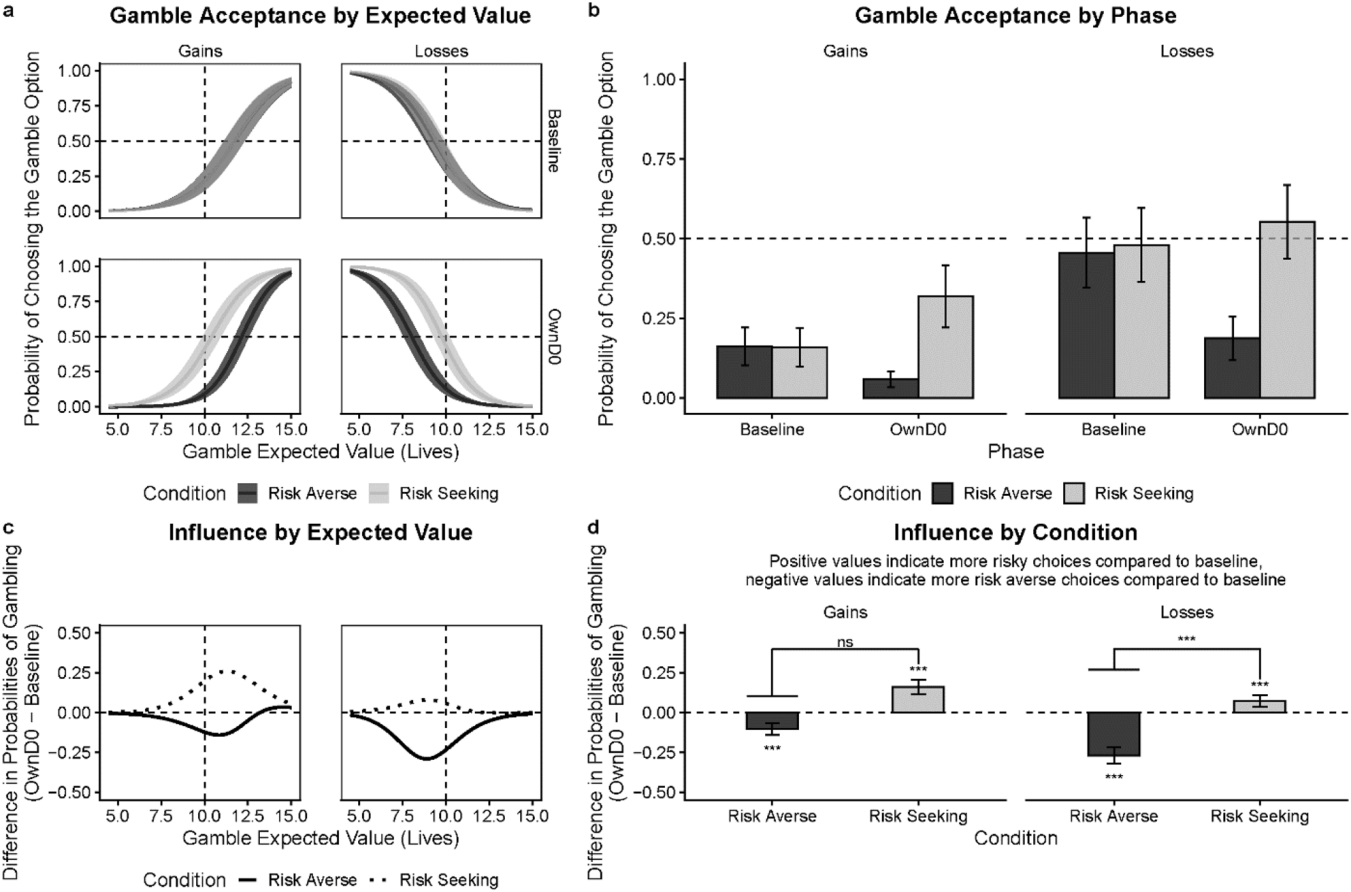
Conformity in the moral domain. (**a**) Gamble acceptance as function of its expected values in the gain or loss frame by norm conditions (i.e., *risk-averse* or *risk-seeking*) in the *baseline* and *OwnD0* (own choices) phases. Vertical dashed lines represent equal expected values of the certain and gamble options. Horizontal dash lines represent equal probability of choosing the gamble or certain option. (**b**) Marginal probabilities of accepting the gamble option across phases by norm conditions. (**c**) Amount of norm influence as a function of expected values of lives for each condition in the gain or loss frame. (**d**) Marginal amount of norm influence for each condition in the gain or loss frame. The amount of influence is represented as the mean difference in gamble probability between *OwnD0* and *baseline* phases. Hanging horizontal bars represent absolute magnitude of the difference. Results presented are based on the data from individuals who completed the session on day 0 (D0). Error bars and confidence bands represent 95% Confidence Intervals (95% CI). *p < .05, **p < .01, ***p < .001.
3. *Were participants generally* *risk-seeking* or *risk-averse*? In the baseline, we found that participants were overall *risk-averse* (see Fig. 2b). Participants chose to gamble less than 50% of the time, as evidenced by a binomial test (*M* = 0.42, 95% CI [0.41, 0.42]; *p* < 0.001). This demonstrates participants’ overall *risk-averse* tendencies on making decisions involving animal lives.
4. *Were participants’ gambling responses consistent with Prospect Theory (PT)?* Although in this experiment participants were overall *risk-averse* at *baseline* for both gains and losses—which counters PT, we found that participants were relatively more *risk-averse* in the gain than the loss frame—which is consistent with PT. Specifically, participants’ baseline probability to gamble was higher in losses (*M* = 0.43, 95% CI [0.36, 0.50]) compared to gains (*M* = 0.18, 95% CI [0.14, 0.23]), as evidenced by a test of contrasts (*c* = 0.24, 95% CI [0.16, 0.32]; *t* = 5.77; *p* < 0.001). Additionally, we applied computational modeling to the data, and found that participants’ empirical gambling behaviors fitted well to the utility functions based on Prospect Theory (for more details see SI and Fig. S-2).
5. *Is there evidence for conformity effects?* We examined the conformity effect for the first session (day 0) and found that participants conformed to the group norms in both framing conditions (see Fig. 2d). Specifically, in the gain frame participants showed the following pattern. In the risk-averse norm condition, they were less likely to gamble in the *OwnD0* phase (*M* = 0.06, 95% CI [0.03, 0.08]) than in the *baseline* (*M* = 0.16, 95% CI [0.10, 0.22]; *c* = −0.10, 95% CI [−0.14, −0.07]; *t* = -5.32; *p* < 0.001). In the *risk-seeking norm* condition participants were more likely to gamble in the *OwnD0* phase (*M* = 0.32, 95% CI [0.22, 0.42]) compared to the *baseline* (*M* = 0.16, 95% CI [0.10, 0.22]; *c* = 0.16, 95% CI [0.12, 0.21]; t = 6.93; *p* < 0.001). We observed the same pattern in the loss frame. In the *risk-averse norm* condition, participants were less likely to gamble in the *OwnD0* phase (*M* = 0.19, 95% CI [0.12, 0.26]) than in the *baseline* (*M* = 0.46, 95% CI [0.35, 0.57]; *c* = −0.27, 95% CI [−0.32, −0.22]; *t* = −10.25; *p* < 0.001). In the *risk-seeking norm* condition, they were more likely to gamble in the *OwnD0* phase (*M* = 0.55, 95% CI [0.44, 0.67]) than in the *baseline* (*M* = 0.48, 95% CI [0.36, 0.60]; *c* = 0.07, 95% CI [0.03, 0.11]; *t* = 3.75; *p* < 0.001). As evidenced by a test of contrasts, in gains the absolute effect of risk-averse norms was much more pronounced than risk-seeking norms (*c* = 0.20, 95% CI [0.13, 0.26]; *t* = 6.01, *p* < 0.001). Interestingly, in gains there were no differences between the absolute size of the impact of different norm types (*c* = −0.06, 95% CI [−0.12, 0.00]; *t* = −1.90, *p* = 0.057). This can be seen in Fig. 2d.
6. *Do conformity effects persist over time?* Next, we examined whether conformity effects persisted after a time delay. As previously mentioned, to this end a separate logit model was fitted to the data collected from participants who completed both sessions. Persistence of the conformity effect was assessed as the relative change in behavior between baseline in the D0 session and their own choices in the D3 session (see Fig. 3). For the *risk-averse* in gains condition, when participants were tested again three days after, their probabilities to gamble were lower in the *OwnD3* phase (*M* = 0.08, 95% CI [0.03, 0.12]) compared to their *baseline* (D0) responses (*c* = −0.18, 95% CI [−0.27, −0.08]; *t* = −4.60; *p* < 0.001). For the *risk-seeking* in gains condition, their gamble probabilities were higher in the *OwnD3* phase (*M* = 0.46, 95% CI [0.30, 0.62]) than *baseline* responses (*c* = 0.26, 95% CI [0.16, 0.35]; *t* = 6.94; *p* < 0.001). Likewise, after a three-day delay, in the loss frame, participants’ gamble probabilities were lower in the *risk-averse* condition (*M* = 0.11, 95% CI [0.05, 0.18]; *c* = −0.30, 95% CI [−0.41, −0.18]; *t* = -6.44; *p* < 0.001) and higher in the *risk-seeking* condition (*M* = 0.58, 95% CI [0.42, 0.73]; *c* = 0.11, 95% CI [0.03, 0.19]; *t* = 3.39; *p* = 0.004). A test of contrasts showed that the effect of the social norm was more pronounced in the risk-averse condition in losses (*c* = 0.11, 95% CI [0.01, 0.21]; *t* = 2.21, *p* = 0.027), but there were no differences between conditions in gains (*c* = 0.01, 95% CI [−0.10, 0.12]; *t* = 0.13, *p* = 0.897). These results demonstrate that social influence on moral decisions persists following a delay of at least three days.

**Figure 3:**
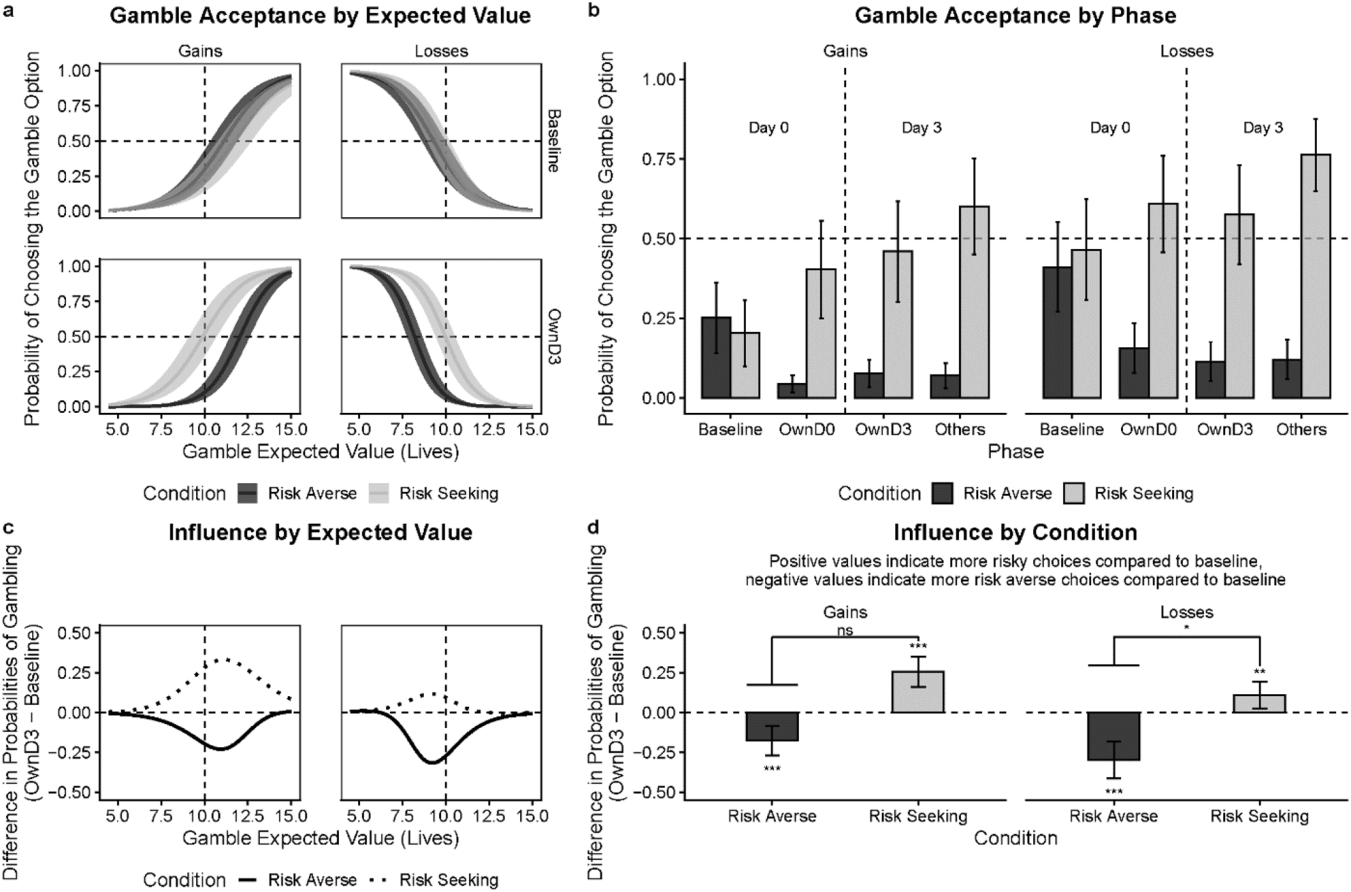
Persistence of conformity in the moral domain. (**a**) Gamble probabilities as function of expected values of lives in the gain or loss frame by norm conditions (i.e., *risk-averse* or *risk-seeking*) in the *baseline* and *OwnD3* (own choices) phases. Dashed lines represent equal expected values of the certain and gamble options (vertical), or the equal probability of opting to gamble or not (horizontal). (**b**) Marginal probabilities of accepting the gamble option across phases by conditions. (**c**) Amount of influence as a function of expected values of lives for each norm condition in the gain or loss frame. (**d**) Marginal amount of influence for each norm condition in the gain or loss frame. The amount of influence is represented as the mean difference in gamble probability between *OwnD3* and *baseline* phases. Hanging horizontal bars represent absolute magnitude of the difference. Results presented are based on individuals who completed both sessions: day 0 (D0) and day 3 (D3). Error bars and confidence bands represent 95% Confidence Intervals (95% CI). *p < .05, **p < .01, ***p < .001.

One concern about the current paradigm is whether the observed conformity effects are driven by demand characteristics: participants may intentionally choose the options which they think represent the group choices to “meet” the experimenters’ expectations, rather their own beliefs. However, we saw little hint of this in debriefing as participants' answer to the question "*To what degree are your choices influenced by other people's choices?"* indicates a fair amount of decision independence (mean = 31.66 on a 0–100 scale, *SD* ± 24.48, where 0 indicates “not at all” and 100 indicates “strongly influenced”). Furthermore, we found that after a three-day delay, participants’ choices for self were different from their choices for guessing others’ preferences (see Fig. 3b). This differentiation suggests that participants' conformity is not the result of demand characteristics or simple parroting of group choices.

One account for normative influence on decision-making is that individuals integrate information learned from the group norms to resolve personal uncertainty during decision-making^38,39^. To examine whether following social norms reduce uncertainty in making moral decisions, we looked at the extent to which participants were consistent in their repeated choices between the same choice options. Our assumption here is that participants who are more certain will be more likely to choose the same option, given repeated identical choices. Technically, we used an entropy measure^40^. The entropy measure is valued between 0 and 1, with higher value indicating more uncertainty in decisions. Specifically, we compared the entropy values between *baseline* and *transfer* phases by fitting binary entropy function to each participant’s gamble probabilities (pair-wise comparison for each choice option). The entropy function is as follows.

$$Entropy = \, - \left( {{\text{Gamble}}} \right)\log_{{2}} p\left( {{\text{Gamble}}} \right) - \left( {{1} - p\left( {{\text{Gamble}}} \right)} \right)\log_{{2}} \left( {{1} - p\left( {{\text{Gamble}}} \right)} \right)$$

We found that participants’ responses in the *baseline* had higher average entropy values than the *transfer* (OwnD0) phase (Wilcoxon sign rank test, *p* < 0.0001). This indicates that after learning about others’ choices, participants alternated less frequently between options, thus reducing the uncertainty in making their own choices. Moreover, we found that the strongest conformity effects occur in situations where participants were most uncertain between the two options. Specifically, the expected values that correspond to the strongest conformity effects match with those with gamble probabilities of 0.5 (most uncertain choices) in the baseline (see Fig. 2a, c). Lastly, although participants overall doubted that others’ choices had a strong impact on their own choices in the debriefing, their self-reported awareness of the norm influence was positively correlated with the degree they conformed in their choices (*r*(358) = 0.19, *p* < 0.001). All these findings suggest that people use information derived from others’ preferences to construct their own preferences. This strategy may be especially helpful for individuals to evaluate decision options when their uncertainty level is high.

### Experiment 2

Experiment 1 showed that participants’ moral risk preferences conformed to the group norms, and conformity effects persisted after three days. In Experiment 2, we extended the scope of the investigation from the moral to the monetary (non-moral) domain. We explored whether participants’ monetary risk preferences would conform to the group’s preferences in gain and loss frames. Similar to Experiment 1, to address the question of norm internalization, we investigated whether conformity effects on monetary choices persisted after three days.

### Participants

371 participants were recruited to complete two experimental sessions from the UCSD’s online psychology subject pool for course credits (262 women; age range = 17–43 years, mean age = 20.49 years, *SD* ± 2.20 years). Both sessions took between 30 min to 1 h to complete, and participants received one separate course credit for doing each session. Similar to the design in Experiment 1, participants were randomly assigned to gain or loss frame and risk-averse or risk-seeking group norm conditions. For the first session at day 0 we excluded 87 participants who failed comprehension checks. One additional individual was excluded due to giving the same answer to all questions in the task. On day 3, 211 participants came back for the second session. We excluded those who failed attention checks or giving the same answer on day 0, leaving us a total of 162 individuals. Out of these individuals, nine were excluded due to failure of comprehension checks, leaving 153 participants (114 female; age *M* = 20.1, *SD* = 1.46). Again, we found no evidence that those who completed both sessions differed from those who only completed the first session (see SI for details).

### Behavioral task

We investigated the conformity effect on participants’ monetary risk preferences using a modified ADP—Money Gamble Task. Similar to the task trial in Experiment 1, participants chose between a certain option with a value of 10, and a gamble option with values ranging from 10 to 30 at a probability of 50%. In the beginning of the procedure, participants were presented with an instruction of either a gain or loss frame of the task as follows:

Welcome to the Gambling Game! The purpose of this research is to understand people’s risk preferences in relation to decisions on monetary gambling. In addition to providing your own opinions, you will be asked to guess how the majority people would respond to various decisions.

Imagine that you are playing a gambling game. You are facing a dilemma where you have to choose between two choices. On each trial, you will be asked to choose between TWO options: Gamble Option—50% chance of losing (gaining) the specified amount of money and: Certain Option—certainly losing (gaining) $10. The amount of money for the Gamble Option will vary across the trials. The amount of money for the Certain Option will stay the same across the trials. You will be asked to indicate your own choice and to guess what the majority people choose.

Then, participants were asked four comprehension questions similar to those in Experiment 1.

### Testing procedure

Participants followed the same procedure described in Experiment 1. We constructed feedback for the group norms presented in the day 0 learning phase based on a pilot study (see SI for more details).

## Results and discussion

Like in Experiment 1, we fitted a mixed effects logit model to participants’ choices. The model included gamble value, experimental phase (*baseline*; *OwnD0*, own choices at day 0 after learning about group norms; *OwnD3*, own choices at day 3; *others*, guessing of others’ choices at day 3), condition (*risk-averse* or *risk-seeking*), and framing (*gains* or *losses*)—with random subject-level intercepts. Separate models were fitted to investigate basic conformity effects and their persistence using data from participants who completed day 0 session and participants who completed both sessions, respectively. In our analysis, we first used the sample from the day 0 session to examine the basic data properties in the *baseline* phase and conformity effects. Further, we examined the persistence of conformity effects using data from those who completed both sessions. Results are summarized below:

1. *Were there any initial baseline differences between groups later assigned to experimental conditions?* Similar to Experiment 1, there were no differences in the *baseline* gamble probabilities between the later-manipulated *risk-averse* and *risk-seeking* conditions in the gain frame (*c* = −0.05, 95% CI [−0.16, 0.06]; *t* = −0.85; *p* = 0.395) or in the loss frame (*c* = −0.01, 95% CI [−0.15, 0.12]; *t* = −0.18; *p* = 0.861). In short, our random assignment was successful.
2. *Were participants sensitive to the expected values? *As seen in Fig. 4a, participants were sensitive to the expected values in the gamble option. They gambled more often as the expected values increased in gains, and less often as the expected values decreased in losses. Consistently with Experiment 1, there was a significant effect of gamble value in gains (*β* = 0.86, 95% CI [0.83, 0.89]; *p* < 0.001) as well as in losses (*β* = −0.55, 95% CI [−0.58, −0.53]; *p* < 0.001).

**Figure 4:**
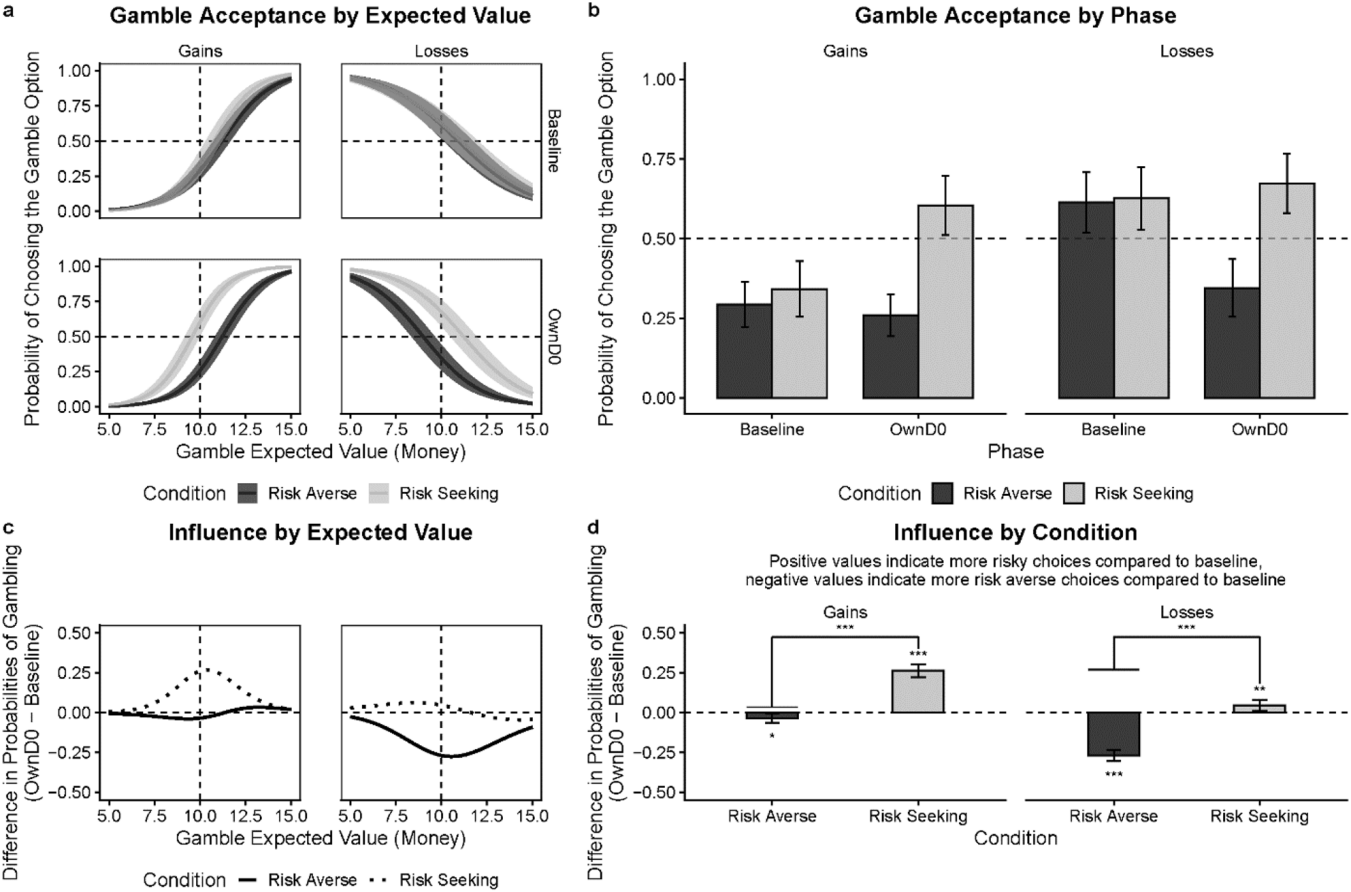
Conformity in the monetary domain. (**a**) Gamble probabilities as function of expected values in the gain or loss frame by norm conditions (i.e., *risk-averse* or *risk-seeking*) in the *baseline* and *OwnD0* (own choices) phases. Dashed lines represent equal expected values of the certain and gamble options (vertical), or the equal probability of opting to gamble or not (horizontal). (**b**) Marginal probabilities of accepting the gamble option across phases by conditions. (**c**) Amount of influence as a function of expected values for each condition in the gain or loss frame. (**d**) Marginal amount of influence for each norm condition in the gain or loss frame. The amount of influence is represented as the mean difference in gamble probability between *OwnD0* and *baseline* phases. Hanging horizontal bars represent absolute magnitude of the difference. Results presented are based on individuals who completed the session on day 0 (D0). Error bars and confidence bands represent 95% Confidence Intervals (95% CI). *p < .05, **p < .01, ***p < .001.
3. *Were participants risk-seeking or risk-averse?* Like Experiment 1, participants were overall *risk-averse* as they opted to gamble less than 50% of the time in the *baseline* (Fig. 4b). This was evidenced by a binomial test (*M* = 0.48, 95% CI [0.47, 0.49]; *p* < 0.001).
4. *Were participants’ gambling responses consistent with Prospect Theory?* Yes, participants’ risk tendencies were fully consistent with Prospect Theory. First, we found that at *baseline* they were more *risk-seeking* in losses (*M* = 0.65, 95% CI [0.57, 0.73]) than in gains (*M* = 0.27, 95% CI [0.21, 0.33]), as supported by a test of contrasts (*c* = 0.38, 95% CI [0.28, 0.48]; *t* = 7.58; *p* < 0.001). Second, as predicted by Prospect Theory that participants were generally risk-seeking (gambled more than 50% of the time) in the loss domain and risk-averse (gambled less than 50% of the time) in the gain domain. Finally, we found that participants’ gambling behaviors fitted well to the utility functions under Prospect Theory (for more details see SI).
5. *Is there evidence for conformity effects?* Next, we investigated conformity effects in each experimental condition for the gain and loss frames (see Fig. 4d). In the gain frame, participants in the *risk-averse* condition gambled less in the *OwnD0* phase (*M* = 0.26, 95% CI [0.19, 0.32]) compared to the *baseline* (*M* = 0.29, 95% CI [0.22, 0.36]; *c* = -0.03, 95% CI [−0.06, −0.01]; *t* = −2.32; *p* = 0.021). In the *risk-seeking* condition they gambled more in the *OwnD0* phase (*M* = 0.60, 95% CI [0.51, 0.70]) than in *baseline* (*M* = 0.34, 95% CI [0.25, 0.43]; *c* = 0.26, 95% CI [0.22, 0.30]; *t* = 12.91; *p* < 0.001). In the loss frame, participants in the *risk-averse* condition gambled less in the *OwnD0* phase (*M* = 0.34, 95% CI [0.25, 0.44]) compared to the *baseline* (*M* = 0.61, 95% CI [0.52, 0.71]; *c* = -0.27, 95% CI [−0.30, -0.23]; *t* = −15.24; *p* < 0.001) and more in the *risk-seeking* condition in *OwnD0* (*M* = 0.67, 95% CI [0.58, 0.77]) than in *baseline* (*M* = 0.63, 95% CI [0.53, 0.73]; *c* = 0.05, 95% CI [0.01, 0.08]; *t* = 2.62; *p* = 0.009). The effect of the social norm (risk-averse vs. risk-seeking norms) was more pronounced in the risk-seeking condition in gains (*c* = -0.23, 95% CI [−0.28, −0.18]; *t* = −9.06, *p* < 0.001) and in the risk-averse condition in losses (*c* = 0.22, 95% CI [0.17, 0.27]; *t* = 8.83, *p* < 0.001), as evidenced by a test of contrasts (Fig. 4d).
6. *Do conformity effects persist over time?* Then, we examined whether the conformity effects persisted after a three-day delay. As shown in Fig. 5, we compared participants’ gamble probabilities between *baseline* and *OwnD3* phases. In gains, participants gambled less in the *risk-averse* condition in the *OwnD3 phase* (*M* = 0.26, CI [0.18, 0.34]; *c* = −0.07, 95% CI [−0.12, −0.02], *t* = −3.68, *p* < 0.001) and more in the *risk-seeking* condition in the *OwnD3* phase (*M* = 0.67, CI [0.56, 0.78]; *c* = 0.27, 95% CI [0.20, 0.35], *t* = 9.21, *p* < 0.001) compared to *baseline*. In losses, participants gambled less in the *risk-averse* condition in the *OwnD3* phase (*M* = 0.44, 95% CI [0.31, 0.57]) compared to *baseline* (*c* = -0.17, 95% CI [−0.23, −0.10]; *t* = −6.83, *p* < 0.001), but did not gamble any more in the *risk-seeking* condition in the *OwnD3* (*M* = 0.68, CI [0.57, 0.79]) relative to *baseline* (*c* = −0.004, 95% CI [−0.07, 0.06]; *t* = −0.15, *p* = 0.999). The absolute effect of the social norm was more pronounced in the risk-seeking condition in gains (*c* = −0.23, 95% CI [−0.30, −0.16]; *t* = -6.61, *p* < 0.001) and in the risk-averse condition in losses (*c* = 0.23, 95% CI [0.16, 0.29]; *t* = 6.68, *p* < 0.001). The participants’ own choices also appeared to differ from their choices for others (see Fig. 5b), which reduces the concern that the conformity effects were the mere reflection of parroting of group choices.

**Figure 5:**
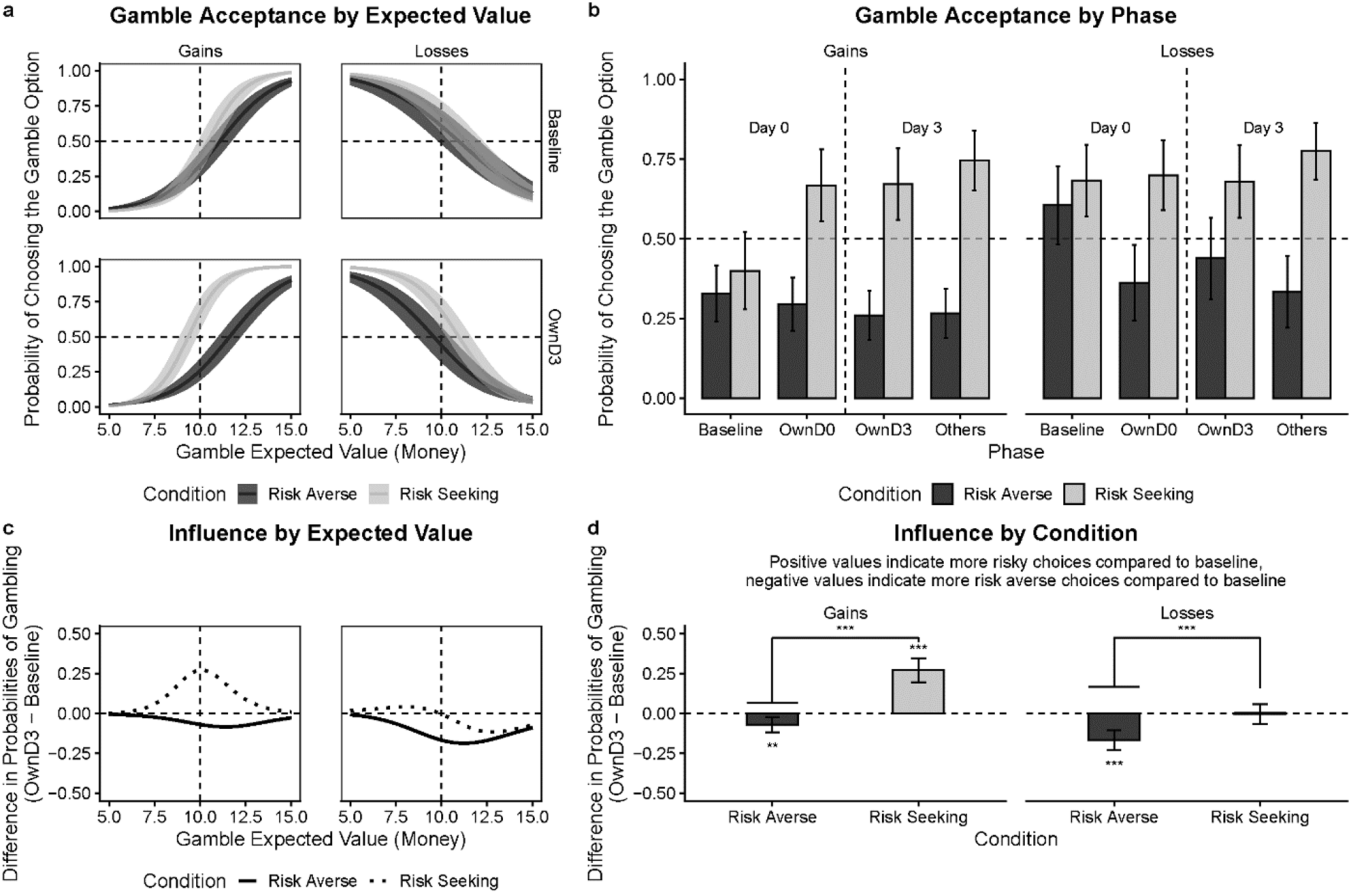
Persistence of conformity in the monetary domain. (**a**) Gamble probabilities as function of expected values in the gain or loss frame by conditions (i.e., *risk-averse* or *risk-seeking*) in the *baseline* and *OwnD3* (own choices) phases. Dashed lines represent equal expected values of the certain and gamble options (vertical), or the equal probability of opting to gamble or not (horizontal). (**b**) Marginal probabilities of accepting the gamble option across phases by conditions. (**c**) Amount of influence as a function of expected values for each condition in the gain or loss frame. (**d**) Marginal amount of influence for each condition in the gain or loss frame. The amount of influence is represented as the mean difference in gamble probability between *OwnD3* and *baseline* phases. Hanging horizontal bars represent absolute magnitude of the difference. Presented are results based on the data collected from individuals who completed the sessions on day 0 (D0) and day 3 (D3). Error bars and confidence bands represent 95% Confidence Intervals (95% CI). *p < .05, **p < .01, ***p < .001.

Following the method of the entropy measure described in Experiment 1, we estimated participants’ uncertainty about choice options. We found that participants reduced uncertainty in making their own choices after learning about others’ choices (Wilcoxon sign rank test, *p* = 0.002). Furthermore, we found that for the experimental conditions where the conformity effects are significant—risk-seeking in gains and risk-averse in losses, the strongest effects occur in the expected values that correspond to the most uncertain choices (baseline gamble probabilities of 0.5; see Fig. 4a and c). Finally, similar to Experiment 1, participants overall did not think that other people's choices had a big impact on their own decisions (mean = 35.07 on a 0–100 scale, *SD* ± 25.05, where 0 indicates “not at all” and 100 indicates “strongly influenced”). However, we found that their awareness of the social influence is positively correlated with their actual conformity behavior (*r*(281) = 0.20, *p* < 0.001). Altogether, these findings suggest that people actively incorporate social information into decision-making under uncertainty.

### Experiment 1 and 2: comparisons between moral and monetary choices

The similar setups for Experiment 1 and 2 allow for direct comparisons between moral and monetary (non-moral) domains. We specifically focus on four aspects of the comparisons: (1) participants’ overall preferences for risk, (2) effects of gain–loss framing, (3) effects of social norms, and (4) degree of conformity effects. To answer these questions, a separate model was fitted to the participants’ choices collected in Experiment 1 and Experiment 2—with the addition of the ADP task version (*moral* or *monetary*) as an explanatory variable. For these analyses, we used data from day 0, so that we can achieve maximal power.

#### Overall risk preferences across domains

**Figure 6:**
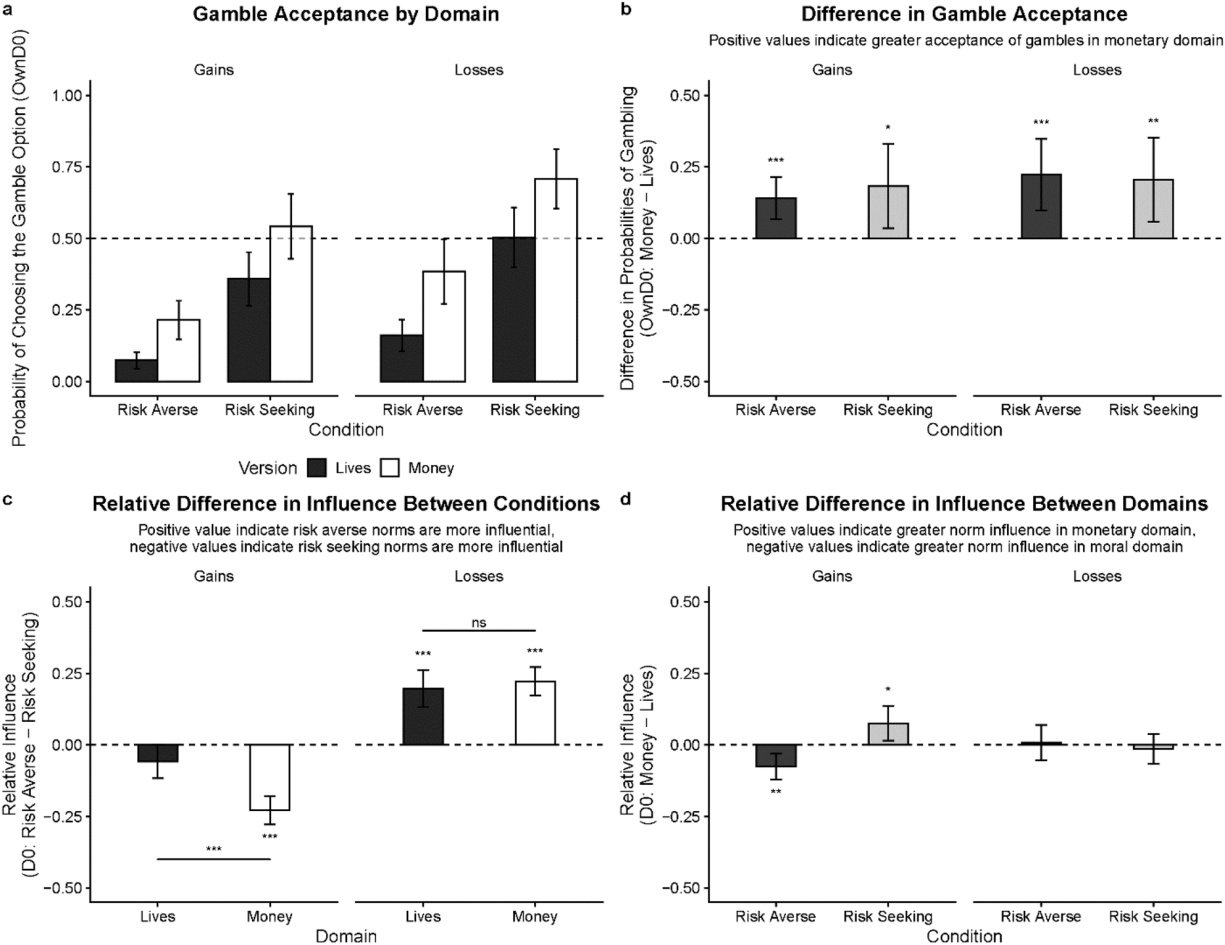
Comparisons between Experiment 1 and Experiment 2. (**a**) Marginal probabilities of endorsing the gamble option in the *OwnD0* phase in the moral (Experiment 1) and monetary (Experiment 2) version of the gamble task. (**b**) Probability differences of endorsing a gamble between moral and monetary versions of the task at the *OwnD0* phase for each experimental condition. (**c**) Differences in the average amount of influence between *risk-averse* and *risk-seeking* conditions. Presented are results based on the data collected from individuals who completed the session on day 0 (D0). (**d**) Differences in the average amount of influence between moral and monetary task versions for each experimental condition. Error bars represent 95% Confidence Intervals (95% CI). *p < .05, **p < .01, ***p < .001.

First, we examined the question whether participants’ risk preferences were different between moral and monetary decisions. We found that overall, so across domains and frames, participants were more risk-seeking (more likely to gamble) with money than lives (Fig. 6a and b), with likelihood differences ranging from 14 to 22. Tests of contrasts (Fig. 6b) revealed differences between moral and monetary domains in mean gamble acceptance in gains (*risk-averse*: *c* = 0.14, 95% CI [0.07, 0.21]; *t* = 3.78, *p* < 0.001; *risk-seeking*: *c* = 0.18, 95% CI [0.04, 0.33]; *t* = 2.44, *p* = 0.015) and in losses (*risk-averse*: *c* = 0.22, 95% CI [0.10, 0.35]; *t* = 3.50, *p* < 0.001; *risk-seeking*: *c* = 0.21, 95% CI [0.06, 0.35]; *t* = 2.73, *p* = 0.006).

One speculation for the lower rates of gambling in the moral domain is inspired by the original PT paper^41^ which suggests that risk-seeking may be reduced by concerns about catastrophic outcomes. We suspect that participants were more concerned with the potential catastrophic outcomes with pandas (an endangered animal) than with money, so they were more prone to avoid gambling with pandas’ lives (since choosing the certain option ensures that only 10 pandas are lost, or at least 10 pandas are saved). This speculation should be addressed in future research, especially because the original version of ADP task did not observe an unusually high degree of risk aversion when dealing with human lives^21,42^.

#### Framing effect across domains

Next, we tested whether the effects of gain–loss framing in the moral decisions show the same pattern seen in the monetary decisions. Our analyses revealed two interesting results. First, in both domains, participants were *relatively* more risk-seeking in losses than gains for both moral and monetary decisions (see “Results and Discussion” sections). This is in line with Prospect Theory assumption that people are relatively more risk-seeking in losses than gains. Note, however, participants' baseline gambling rates in the moral domain are always below 50% (risk aversion), even in the loss domain (Fig. 2b). In the monetary domain, participants baseline results revealed that they were truly risk-seeking in the loss domain (gambling over 50%) and truly risk-averse in the gain domain (gambling below 50%) as shown in Fig. 4b. Second, as mentioned, the effects of framing were significant and sizable in both domains. Note that in the baseline phase, we observed 38% greater likelihood of gambling in losses vs gains in the monetary domain (65% vs 27%), and 24% greater gambling in losses than gains in the moral domain (42% vs 18%). As evidenced by a test of contrasts, the difference in gamble probabilities between gains and losses in the baseline phase was greater in the monetary vs moral domain (*c* = 0.14, 95% CI [0.01, 0.27]; *t* = 2.12, *p* = 0.034). Though this difference in the size of the framing effect was significant, it is hard to interpret since, as mentioned, participants were overall more risk-averse in the moral domain and had less room to move.

#### Influence of social norms across domains

Third, we examined the influence of different social norms (risk-averse vs risk-seeking) for moral and monetary decisions. Shown in Fig. 6c, we found that risk-averse norms have more influence than risk-seeking norms on participants’ gambling preferences in the loss frame. This is true for the moral domain: *c* = 0.20, 95% CI [0.13, 0.26]; *t* = 6.01, *p* < 0.001; and the monetary domain: *c* = 0.22, 95% CI [0.17, 0.27]; *t* = 8.83, *p* < 0.001). In contrast, risk-seeking norms have more influence in the gain frame. This, however, was true only for the monetary domain: c = −0.23, 95% CI [−0.28, −0.18]; t = −9.07, p < 0.001), but not in the moral domain: *c* = −0.06, 95% CI [−0.12, 0.00]; *t* = −1.90, *p* = 0.057, where the influence of risk-seeking and risk-averse norms was comparable. This relative difference in influence of different norms across domains was only observed in gains (*c* = 0.15, 95% CI [0.07, 0.23]; *t* = 3.89, *p* < 0.001), but not in losses (*c* = −0.02, 95% CI [−0.10, 0.06]; *t* = −0.55, *p* = 0.584).

#### Conformity effect across domains

Finally, we compared the relative degree of conformity in monetary vs. moral decisions across different experimental norm conditions (Fig. 6d). In the gain fame, we found that risk-averse norms have more influence in the moral domain than in the monetary domain (*c* = −0.08, 95% CI [−0.12, −0.03]; *t* = −3.24, *p* = 0.001). In contrast, risk-seeking norms have more influence in the monetary domain than in the moral domain (*c* = 0.08, 95% CI [0.01, 0.14]; *t* = 2.43, *p* = 0.015). However, in the loss frame, differences in conformity between moral and monetary domains were not significant for either *risk-averse* (*c* = 0.01, 95% CI [−0.05, 0.07]; *t* = 0.25, *p* = 0.805) or *risk-seeking* (*c* = −0.01, 95% CI [−0.07, 0.04]; *t* = −0.56, *p* = 0.557) norm conditions. We note that these results are not due to participants’ selectively poor norm learning in some experimental conditions as participants across all conditions exhibited good accuracy in learning about the group norms (see SI for details).

### General discussion

Taken together, this research examined social influence on individuals’ risk preferences in different domains and contexts. We used a modified ADP that involved scenarios with gain or loss framing, in moral or monetary domains, and exposed participants to risk-seeking or risk-averse group norms. Overall, we found that for decisions about both lives and money, people shifted their risk preferences towards the group norm. This effect persisted over three days suggesting some evidence of norm internalization, rather than responding to outward pressure.

Importantly, we also found that conformity effects depend on the types of social norms, framing of the choice, and decision domain. One pattern we observed is that in the monetary domain the norms were more influential when they ran against participants' default behavior, which, in the baseline condition conformed to Prospect Theory. That is, whereas at baseline participants were risk-averse for gains and risk-seeking for losses, the risk-seeking norm was more influential in the gain domain and the risk-averse norm was more influential in the loss domain. Theoretically, this suggests that in the monetary domain social norms play an informational function—they are more surprising, and hence influential, when they go against assumptions about what most people do in a similar situation^43^. Feedback is likely to be most surprising when it violates not only *quantitative*, but also *qualitative* expectations about the overall direction of risk attitudes (i.e., whether people are risk-averse or risk-seeking on the whole). For example, if one expects the majority to be risk-averse in the gain domain, observing that majority is risk-seeking may have larger effects on belief and behavior (whereas merely observing that people are more risk-averse than one expected may have smaller effects).

In the moral domain, the pattern of norm influence was more subtle. In the loss frame, risk-averse norms had bigger impact than risk-seeking norms, similar to what we found in monetary domain. However, in the gain frame, the impact of risk-seeking and risk-averse norms was more uniform. There are several possible explanations as to why in the present research conformity in the moral domain is more uniform, at least in gains (i.e. when the task is framed as saving pandas). First, conformity in the moral domain may be compelled by both descriptive norms (what most people do) and injunctive norms (what most people should do)^29^. In contrast, as mentioned, in the monetary domain, the norms may be primarily descriptive, lessening the normative pressure and functioning primarily as information about typical behavior. Second, our moral domain involved decisions for others (choosing for pandas) whereas our monetary domain involved decisions for self. It is possible that we found more uniform influence of the group norms in the moral domain because these decisions were for others, and were about saving (perhaps triggering more associations with prosocial actions). This is related to the emphasis in the literature that moral decisions are tied to reciprocity norms and group acceptance^28,31^. Third, moral conformity is related to the desire to maintain good reputation^30^. In addition, participants may want to avoid responsibility for wrong choices related to saving pandas' lives, which may in turn lead to conformist behaviors by following others’ choices in order to eventually diffuse moral responsibility for the outcomes^44,45^.

Another interesting general pattern observed in our research was the relatively greater aversion to risk in the moral domain than the monetary domain. In fact, even in the loss frame (which typically promotes risk-seeking) the gambling rates with lives remained below 50%. As mentioned earlier, participants could be overall reluctant to gamble (i.e., risk a large loss of lives, or not saving any lives) when only a limited total number of individuals exists in the population. In the language of decision-making, this risk aversion (preference for a certain smaller than a probabilistic larger loss) could indicate *greater* sensitivity to larger than smaller losses, which is a boundary condition acknowledged in Prospect Theory and should primarily occurs when losses are near catastrophic^41^. Alternatively, participants could avoid the risky loss of more lives because thinking about losses highlights concerns of mortality^46^. This risk-averse attitude may also reflect deontological considerations such as a societal prescription not to “gamble with death” or not to treat live or death decisions like a “Russian Roulette”^47^. These considerations may also explain why risk-averse norms had more influence on decisions about saving lives than about earning money.

The current research has several limitations, and points to numerous future directions. To construct the feedback for social norms, we used the group average gamble rates from a pilot study. Future studies can construct the norms to be tailored to the individual participant’s preferences, as this can provide a more precise measure of the norm influence. Studies can examine the dependency of individuals’ risk preferences on norm influence. For example, do individually risk-averse participants conform more when the perceived group norm is also risk-averse, and vice versa^48^? Moreover, because the nature of our feedback was probabilistic (see SI for details), future research can address the issue of group norm consistency by creating non-probabilistic feedback. Another factor to consider is the exact nature of the group providing the norm. In our research, participants most likely presume that they were getting information about their undergraduate peers, so the key norm driving our observed effects comes from the peer group. This is consistent with previous research that people are more influenced by local norms (e.g., peer norms) than global norms (non-peer norms)^49^ and people who self-identify with the group exhibit greater conformity^50^. Thus, future research can further test the effects of norms of different groups, and different cultures with varying emphasis on group adherence^51^. A related direction for future research involves identifying conditions under which people will conform to, resist, or act against social norms (e.g., reactance phenomena). For example, future work can explore whether different ways of delivering normative information result in differential conformity effects. Previous research suggests that a descriptive normative message may backfire and create boomerang effects, whereas adding an injunctive message can eliminate these undesirable effects^52^. It is also unknown whether the conformity effects extend to other domains of moral decision-making, and to other ways of structuring the decision, so that it involves the self. For example, is conformity different when people are making risky decisions whether to sacrifice their own time for charity? This is important since research shows that when people make financial decisions for themselves, as compared to for others, they are more risk-averse in gains and more risk-seeking in losses^53^.

Furthermore, our analysis with entropy measure suggests that people incorporate information from the social norms to resolve uncertainty in decisions. This can be formally examined in future work in a neurocomputational framework^39^, and by experimentally manipulating certainty of one’s own preferences and the internal variability of the group. A more general limitation, common to most experimental studies on moral, medical, and financial decision-making, is the hypothetical nature of our choices. Studies that compared real and hypothetical moral choices point out to limitations of hypothetical paradigms^54^. Similarly, studies that compared real and hypothetical financial decisions suggests that real choices are sometimes associated with more loss aversion, especially when stakes are high^55,56^. It is worth noting though that the Asian Disease Paradigm (ADP) used in the current research has been very successful in identifying mechanisms underlying financial and non-financial choices across multiple domains, and in the real world^42,46,57,58^. Using ADP to understand social conformity can help with designing more effective public policy whenever following social norms might be desirable or undesirable. For example, risky health behaviors, with moral consequences for others, could be positively nudged by social norms, especially in populations that are susceptible to peer pressure^59^. As such, we suggest that the current work provides evidence that people conform to social norms when gambling with lives and money, and highlights common and unique features of decision making and conformity across these domains.

## Supplementary Information

Supplementary Information.

## Data Availability

The data that support the findings of this study are available at https://osf.io/vfg57/.

## Code availability

The experiment script and codes used for analysis are available from the corresponding author upon reasonable request.
