# Publisher Correction: People conform to social norms when gambling with lives or money

**DOI:** 10.1038/s41598-023-30107-y

**Published:** 2023-02-17

**Authors:** Yueyi Jiang, Przemysław Marcowski, Arseny Ryazanov, Piotr Winkielman

**Affiliations:** 1 Department of Psychology, University of California San Diego, La Jolla, CA USA; 2 Swartz Center for Computational Neuroscience, University of California San Diego, La Jolla, CA USA; 3 Department of Psychology, SWPS University of Social Sciences and Humanities, Warsaw, Poland

Correction to: *Scientific Reports* 10.1038/s41598-023-27462-1, published online 16 January 2023

In the original version of this Article, Piotr Winkielman was omitted as a corresponding author. Correspondence and requests for materials should also be addressed to pwinkielman@ucsd.edu.

The original Article has been corrected.

