# Supplementary material for: People conform to social norms when gambling with lives or money
Source: Sci Rep. 2023 Jan 16;13:853. doi: 10.1038/s41598-023-27462-1 (PMC9842616; doi:10.1038/s41598-023-27462-1)
Supplement: Supplementary file 1 — Supplementary Information. [file 41598_2023_27462_MOESM1_ESM.docx]

**Supplementary Materials**

*Risk preference estimates*

To estimate changes in participants’ risk preference, we used a model-based approach by computing risk preference parameter based on Prospect Theory for each participant. The subjective utility associated with gains or losses $x$ is $v\left( x \right),$following the equation below.

$$v(x)=\left\{ \begin{matrix} x^{\alpha} x\geq0 (gain frame) \\ {-\left( -x \right)}^{\beta} x<0(loss frame) \end{matrix} \right.$$

Our risk preference parameters α and ß govern the curvature of the subjective utility functions. A smaller value of α or ß indicates a faster decrease in marginal utility in gains or losses. Thus, specifically, alpha increasing means more risk-seeking in the gain frame, and beta increasing means more risk aversion in the loss frame. Note that the value function within Prospect Theory includes a loss aversion parameter, which is not applicable to this study since no condition with mixed gain-loss gambles was presented. The subjective values for the gamble and certain options are defined by

$$V\left( Gamble \right)=v(x)$$

$V\left( Certain \right)=$ $v\left( \theta\right)$

Where $x$is the outcome for gamble wins or losses, and $\theta$ is the outcome of the certain gains or losses. In this equation, we ignored the probability weighting and set $p\left( 0.5 \right)=0.5.$The probability of taking the gamble option depends on the difference in the expected utility of gamble and certain options following a sigmoid function.

$$p\left( Gamble \right)=\frac{1}{1+exp(\tau\left( V\left( Certain \right)-V\left( Gamble \right) \right))}$$

where $\tau$ is a sensitivity parameter that quantifies the extent to which the choice behavior is determined by the difference in subjective utility for the certain option and the gamble option. When$\tau$ = 0, the gambling probability is 50%. As $\tau$ grows larger, the difference in subjective utility of the two options will result in a more deterministic choice.

*Participant learning accuracy*

To probe whether participants were able to learn the group norms during the learning phase, we calculated the percentage of the correct responses in the last 30 trials as accuracy metrics. Using a bootstrapping approach, we estimated 95% confidence intervals for the accuracy metrics.

*Feedback for the group norms*

For Experiment 1 and 2, we constructed feedback for the group norms based on pilot data. For each experiment, we used a generalized linear model with a logit link and a binomial distribution to predict the probabilities of gambling for the gamble values ranged from 10 to 30 with increments by 2 (the pilot study has different sets of the gamble values tested). We show the model estimates in **Fig. S-3.** Subsequently, we adapted them to make risk-averse group norms (i.e., 20% above group gambling rate) and risk-seeking group norms (i.e., 20% below group gambling rate).

**Supplementary Results**

*Is there a selection bias in the analyses of Day 3 behavior given that some participants from session 1(D0) did not show up for session 2 (D3)?*

In this research, we intentionally advertise this study as two separate sessions (to avoid demand characteristics), and participants could receive one separate course credit for the participation of each study session. Since participants were UCSD students who were doing the study for course credits, and they received one separate course credit for each session, they may not be incentivized to come back for the follow-up session. However, to address a potential issue of selection bias, we tested if participants who showed up for the follow-up session on day 3 differed from those who did not by refitting our models to the data collected in day 0 session -- with an additional variable indicating whether a given individual showed up on day 3 or not.

We observed no significant effect of showing up on day 3 on the overall probability of choosing a gamble over the certain option. There was no difference in Experiment 1 (𝛸^2^ = 0.17, *p* = .680) or in Experiment 2 (*X*^2^ = 0.63, *p* = .429). In addition, we looked at potential differences in conformity – defined as influence of the group norm on their probability of gambling between the baseline and the transfer phase on Day 0. We found no difference between participants who showed up for the follow-up session and those who did not. This was true Experiment 1 (t = -0.34, *p* = 0.731; c = -0.01, 95% CI [-0.05, 0.04]) or Experiment 2 (t = 0.72, *p* = 0.470; c = 0.01, 95% CI [-0.02. 0.05]).

Finally, to assess if individuals who did not show up on day 3 were paying attention to the choice options (a metric for data quality), we tested if they were sensitive to the expected value of the gambles. A simple slope analysis (based on the model mentioned above) showed that they were sensitive to the expected values of the gambles in experiment 1 (𝛽_EV_ = 0.11, 95% CI [0.08, 0.14] and 𝛽_EV_ = -0.19, 95% CI [-0.21, -0.17] in gains and losses, respectively) and experiment 2 (𝛽_EV_ = 0.18, 95% CI [0.16, 0.20] and 𝛽_EV_ = -0.13, 95% CI [-0.14, -0.12] in gains and losses, respectively)

In conclusion, this evidence suggests that there was no difference in the key variables of the experiments (e.g., data quality, participants’ choices, and general tendency to conform) for whether participants showed up for the follow-up session or not.

*Computational modeling analysis*

The model parameters were estimated for each condition and shown in **Table S1** for Experiment 1 (moral domain) and Experiment 2 (monetary domain). In the moral domain, $\alpha$ increased in the risk-seeking condition and decreased in the risk-averse condition. Similarly, $\beta$ decreased in the risk-seeking condition and increased in the risk-averse condition. In the money domain, $\alpha$ increased in the risk-seeking condition and $\beta$ increased in the risk-averse condition. There was no significant change in either risk-averse condition in the gain frame or risk-seeking condition in the loss frame. These results align with the observed conformity effects in the experiments. The utility functions based on the estimated parameters for baseline and transfer phases are presented for all conditions in **Fig. S-1**. Overall, our model fits our data well by minimizing the L2 distance between empirical data and model prediction. The model fitting performance is shown in **Fig. S-2.**

*Participant learning results*

Shown in **Table S2,** participants had higher than chance accuracy in the last 30 trials of the learning phase for all conditions. This indicates that overall participants were able to learn the group norms.

**Table S1 |** Model Parameter Estimates

**Experiment 1 (Moral Domain)**

|  | Gain | | | | Loss | | | |
| --- | --- | --- | --- | --- | --- | --- | --- | --- |
|  | Risk Averse | | Risk Seeking | | Risk Averse | | Risk Seeking | |
|  | *Base* | *Own-D0* | *Base* | *Own-D0* | *Base* | *Own-D0* | *Base* | *Own-D0* |
| α | 0.834 | 0.785 | 0.833 | 0.964 |  |  |  |  |
| β |  |  |  |  | 1.138 | 1.479 | 1.101 | 1.063 |
| $\tau$ | 0.887 | 1.850 | 0.990 | 0.695 | 0.388 | 0.126 | 0.390 | 0.499 |

**Experiment 2 (Monetary Domain)**

|  | Gain | | | | Loss | | | |
| --- | --- | --- | --- | --- | --- | --- | --- | --- |
|  | Risk Averse | | Risk Seeking | | Risk Averse | | Risk Seeking | |
|  | *Base* | *Own-D0* | *Base* | *Own-D0* | *Base* | *Own-D0* | *Base* | *Own-D0* |
| α | 0.869 | 0.866 | 0.914 | 1.085 |  |  |  |  |
| β |  |  |  |  | 0.911 | 1.210 | 0.871 | 0.864 |
| $\tau$ | 0.844 | 1.051 | 0.859 | 0.532 | 0.603 | 0.284 | 0.666 | 0.900 |

Model parameter estimates are presented in both experiments in the *baseline* and *Own-D0* phases. Note: In the gain frame, α increasing means more risk-seeking; in the loss frame, β increasing means more risk-averse.

**Table S2 |** Learning Accuracy

| **Condition** | **Accuracy (Last 30 Trials)** | |
| --- | --- | --- |
|  | **Moral** | **Money** |

| **Risk Averse Norms** | **Gain** | **0.76**  [0.74, 0.78] | **0.67**  [0.65, 0.69] |
| --- | --- | --- | --- |
|  | **Loss** | **0.67**  [0.64, 0.69] | **0.65**  [0.63, 0.67] |
| **Risk Seeking Norms** | **Gain** | **0.67**  [0.65, 0.69] | **0.64**  [0.61, 0.66] |
|  | **Loss** | **0.56**  [0.54, 0.58] | **0.71**  [0.68, 0.73] |

Bold indicate 95% confidence intervals that excluded 0.5 for accuracy after bootstrapping. All conditions exhibited accuracy above chance in both moral and monetary domains.

**Fig S-1**| Utility Functions

***
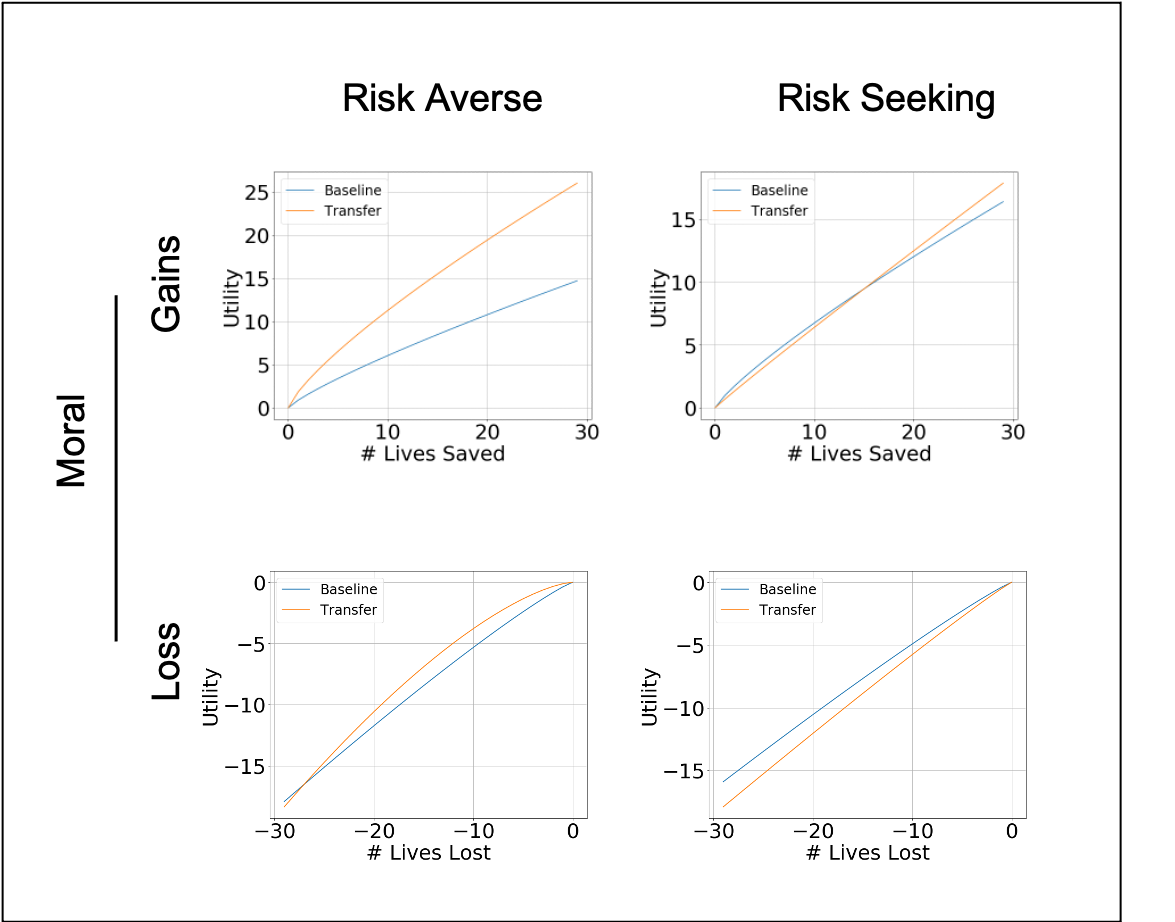
***

***
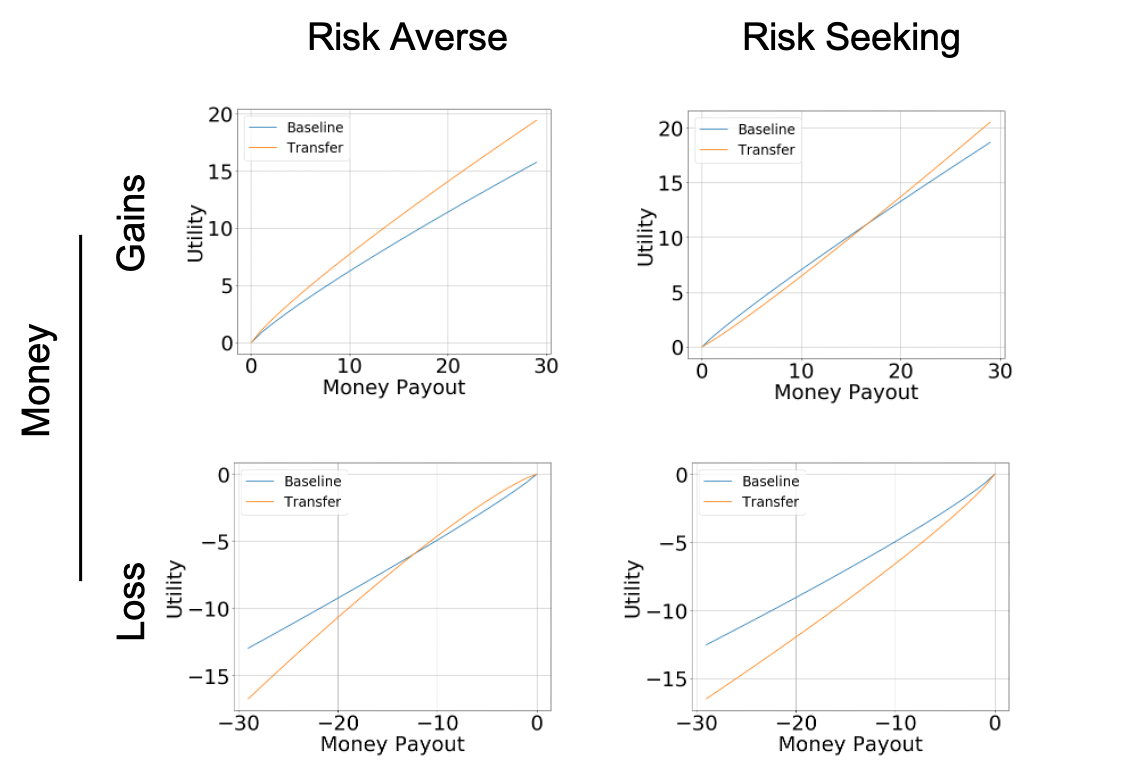
***

The utility functions based on the estimated model parameters for baseline and transfer (*OwnD0*) phases are presented for all conditions.

**Fig S-2**| Model Fitting Performance

***
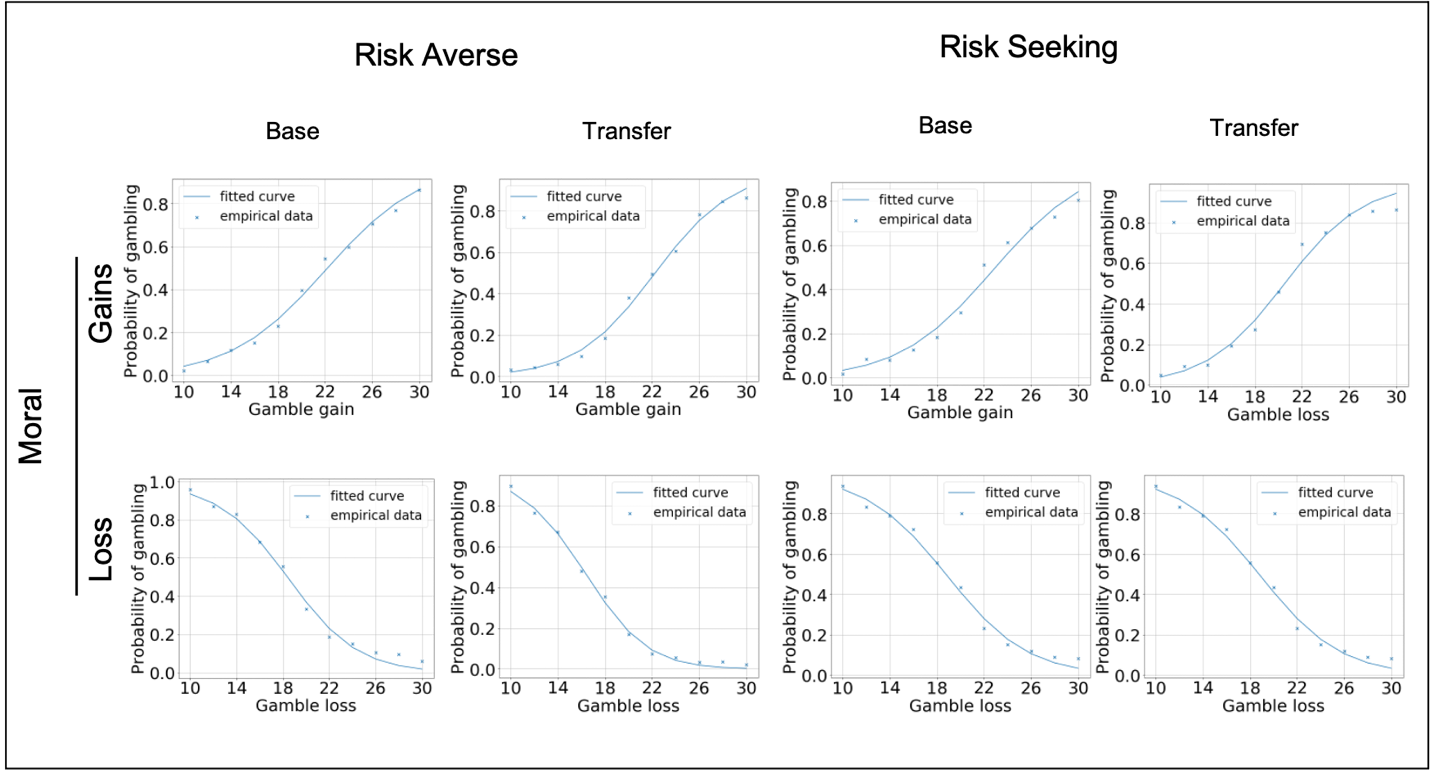
***


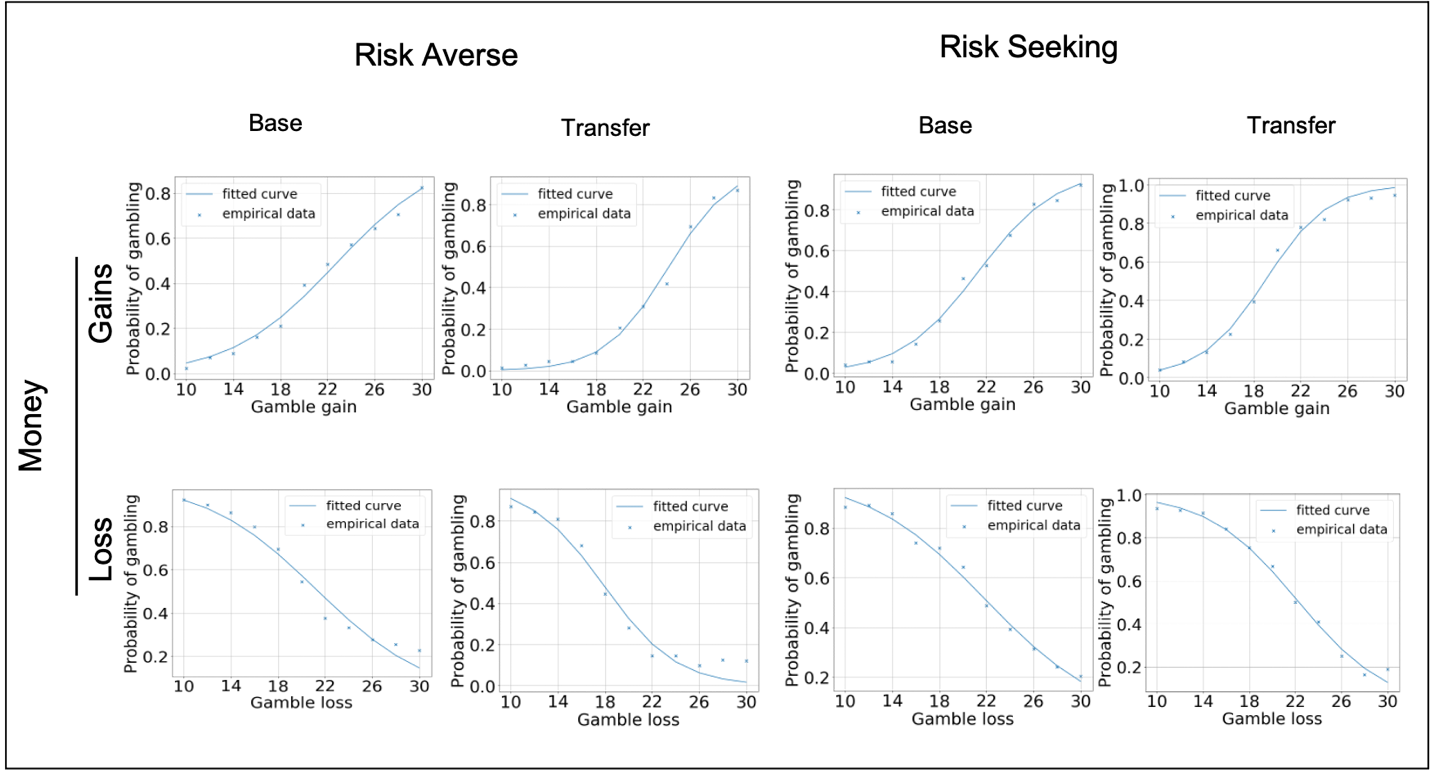


The probabilities of gambling in the baseline and transfer phases are presented for each gamble value. Dotted line represents the empirical data for each condition, and the solid line shows the estimated probabilities from the model fitting.

**Fig S-3** | Group Average Gambling Rates in the Pilot Data


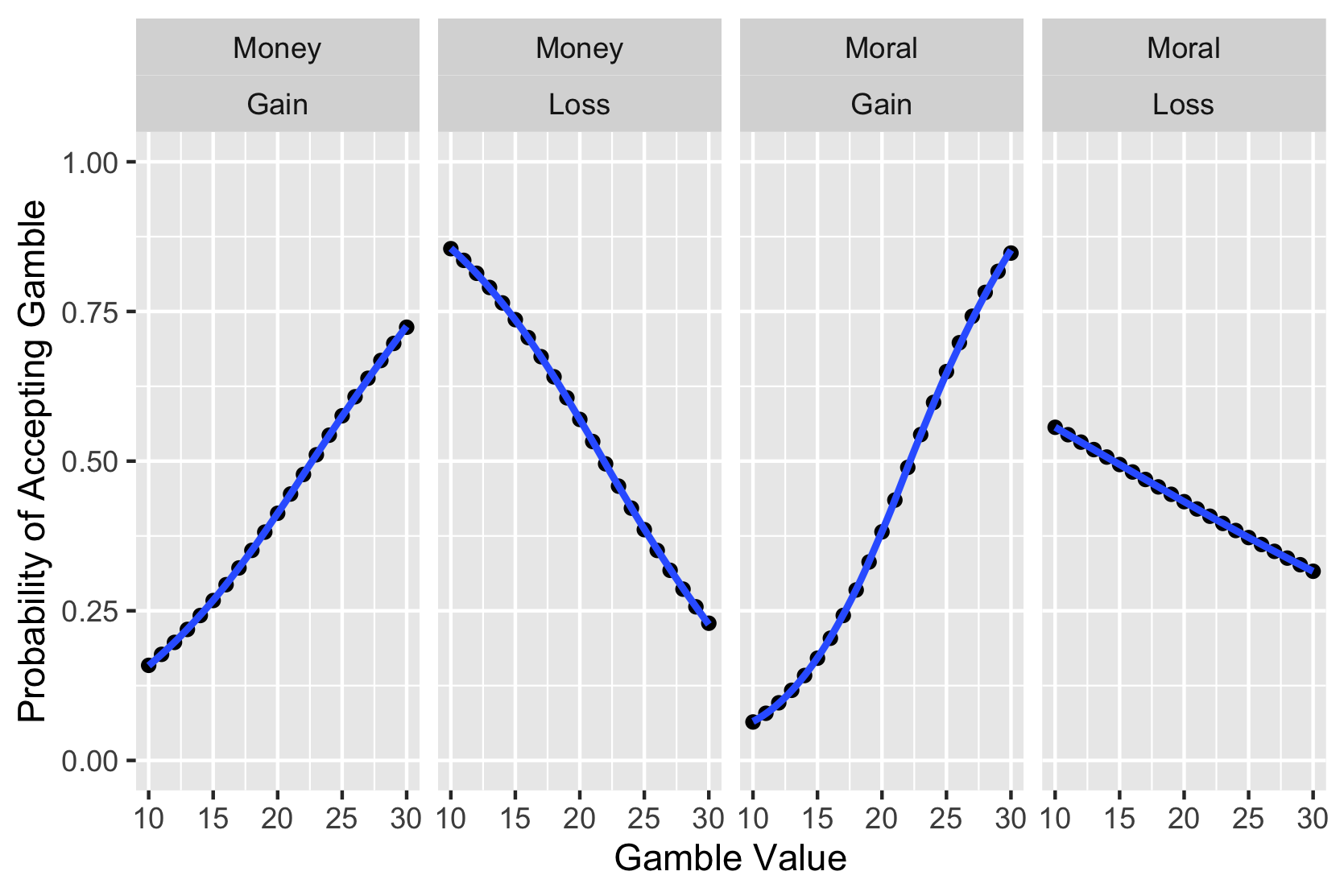


Pilot study data (model estimates) for both money and moral domains. Participants’ group average gambling rates are shown for each value presented in the risky gamble option (2 x EV). For example, gamble value 20 means that EV was equal 10 (20/2).
